# Integrating Nanopore MinION Sequencing into National Animal Health AMR Surveillance Programs: An Indonesian Pilot Study of Chicken Slaughterhouse Effluent and Rivers

**DOI:** 10.3390/antibiotics14070624

**Published:** 2025-06-20

**Authors:** Rallya Telussa, Puji Rahayu, Thufeil Yunindika, Curtis J. Kapsak, Kanti Puji Rahayu, Oli Susanti, Imron Suandy, Nuraini Triwijayanti, Aji B. Niasono, Syamsul Ma’arif, Hendra Wibawa, Lestari Lestari, Gunawan B. Utomo, Farida C. Zenal, Luuk Schoonman, Lee E. Voth-Gaeddert

**Affiliations:** 1 FAO Country Office for Indonesia, Jakarta 10250, Indonesia; rallya.telussa@fao.org (R.T.); luuk.schoonman@fao.org (L.S.); 2 Quality Control and Animal Product Certification Laboratory, Ministry of Agriculture, Jakarta 12550, Indonesia; puji_latif@yahoo.co.id (P.R.);; 3 Theiagen Genomics, Highlands Ranch, CO 80129, USA; 4 Directorate Veterinary Public Health, Ministry of Agriculture, Jakarta 12550, Indonesia; 5 Disease Investigation Center Wates, Ministry of Agriculture, Yogyakarta 55651, Indonesia; 6 Center for Health Through Microbiomes, Biodesign Institute, Arizona State University, Tempe, AZ 85281, USA; 7 Global Health Engineering, LLC, Saint Louis, MO 63116, USA

**Keywords:** *Escherichia coli*, waterborne pathogens, extended-spectrum beta-lactamases (ESBLs), genomic sequencing, Tricycle Protocol

## Abstract

**Background:** Antimicrobial resistance (AMR) poses significant risks to human and animal health, while the environment can contribute to its spread. National AMR surveillance programs are pivotal for assessing AMR prevalence, trends, and intervention outcomes; however, integrating advanced surveillance tools can be difficult. This pilot study, conducted by FAO ECTAD Indonesia and DGLAHS, the Indonesian Ministry of Agriculture, evaluated the costs and benefits of integrating the Nanopore MinION, Illumina MiSeq, and Sensititre system into a culture-based slaughterhouse–river surveillance system. **Methods:** Water samples were collected from six chicken slaughterhouses and adjacent rivers (pre- and post-treatment effluent, upstream, and downstream). Culture-based ESBL and general *E. coli* concentrations were estimated via the WHO Tricycle Protocol, while isolates (*n* = 42) were sequenced (MinION, MiSeq) and antimicrobial susceptibility testing conducted (Sensititre). **Results:** The Tricycle Protocol results provided estimates of effluent and river concentrations of ESBL and general *E. coli* identifying ESBL-to-general *E. coli* ratios of 13.8% and 6.2%, respectively. Compared to hybrid sequencing assemblies, MinION had a higher concordance than MiSeq for ARG identification (98%), virulence genes (96%), and locations for both (predominately plasmids). Furthermore, MinION concordance with Sensititre AST was 91%. **Conclusions:** Cost–benefit comparisons suggest sequencing can complement culture-based methods but is dependent on the value placed on the additional information gained.

## 1. Introduction

Antimicrobial resistance (AMR) is a critical global health issue. The emergence and spread of AMR is complex and occurs through people, animals, and the environment [1]. In 2021, antimicrobial resistance (AMR) was associated with 4.71 million deaths, including 1.14 million directly attributable to AMR, and by 2050, AMR is projected to cause 8.22 million deaths annually, with 1.91 million directly attributable [2]. To fully understand, monitor, and control the dissemination and environmental fate and transport of AMR, a one-health approach—people, animals, and the environment—must be leveraged [3]. Within this complex one-health system (or systems) are critical control points where control strategies can be most effective at curbing AMR transmission [4]. Standardized AMR surveillance systems are key for (1) ensuring robust regional and global comparisons of temporal trends, (2) identifying emerging AMR pathogens to inform clinical responses, (3) supporting locally tailored regulatory frameworks, (4) identifying where control strategies would be most effective and (5) measuring the effectiveness of the control strategy. Standardization includes the sampling and laboratory protocols, the types of AMR indicator bacteria evaluated, and the types of environments (and their critical control points) to monitor [5,6,7].

Three core methods to facilitating AMR surveillance programs include culture-based, qPCR-based, and whole genome sequencing-based (including isolate and metagenomics). These methods are applied and/or integrated in different ways across different one-health systems and geopolitical regions. Two global AMR surveillance system approaches aimed at helping standardize methods include (1) the ESBL *Escherichia coli* (*E. coli*) Tricycle Protocol [8,9] and (2) PulseNet International [10]. The Tricycle Protocol is a proposed, low-cost method that uses a culture-based procedure to estimate the abundance of total *E. coli* and extended-spectrum beta lactamase (ESBL)-producing *E. coli* across the three one-health systems (human health, food chain, and the environment). The PulseNet International protocols use a whole genome sequencing (WGS)-based method to evaluate genetic characteristics of individual isolates (clinical and non-clinical). While understood well in theory, evaluating the practical costs and benefits of integrating the two approaches within the context of institutional program operations is important. This can be conducted by (1) sequencing the isolates from the Tricycle Protocol and (2) validating the use of WGS data for the detection of antimicrobial resistance determinants by comparison to phenotypic Antimicrobial Susceptibility Testing (AST). In addition, understanding differences in results and costs for different sequencing technologies and approaches is important. Comparing outputs from Illumina MiSeq sequencing technology, the Oxford Nanopore MinION sequencing technology, and ‘gold standard’ hybrid assemblies can aid in selecting the optimal technologies and approaches. Finally, bioinformatic tools that do not require command line expertise can allow for the further decentralized adoption of these advanced sequencing technologies but require simplified processing and analysis tools.

To evaluate these methods, this pilot study was designed by the FAO and DGLAHS with the aim of assessing the concentration and genetic characteristics of ESBL *E. coli* in the effluent and receiving river bodies of chicken slaughterhouses in the Greater Jakarta area (Jakarta, Bogor, and Tangerang), Indonesia. Slaughterhouse effluent and rivers are potential locations for monitoring critical control points related to the animal–environment nexus of the one-health system for AMR transmission. ESBL-producing *Enterobacteriaceae* is listed as a critical threat to human health by the CDC AR threats report [11], supporting the use of ESBL *E. coli* as an indicator bacteria to monitor. Furthermore, recent data suggest chickens and associated slaughterhouses can contribute to the transmission of AMR. Day et al. 2019 suggests ESBL *E. coli* infections among humans are increasing and chickens may contribute to the transmission of pathogenic ESBL *E. coli* between chickens and humans [12]. Further data suggest water (effluent and rivers) can be a primary mode of the transmission and spread of ESBL *E. coli* [13,14,15]. In Indonesia, the majority of chicken slaughterhouses are located near rivers as there is often a significant level of effluent created with the slaughtering process (personal communication). Furthermore, slaughterhouses are required by law to treat their effluent before discharging into the environment (the Directorate of Veterinary Public Health, the Ministry of Agriculture, 2021).

In this pilot study, we evaluated the set of methods by sampling and testing slaughterhouse effluent and upstream and downstream river water for six chicken slaughterhouses adjacent to rivers. The questions evaluated in this pilot study included the following:

1. What types and concentrations of *E. coli* are present in the effluent of chicken slaughterhouses and the receiving rivers?
2. Can the Oxford Nanopore MinION sequencing technology provide valid and valuable data considering costs within regional Indonesian AMR monitoring systems currently deploying the Tricycle Protocol?

## 2. Results

### 2.1. E. coli Concentrations

**Figure 1:**
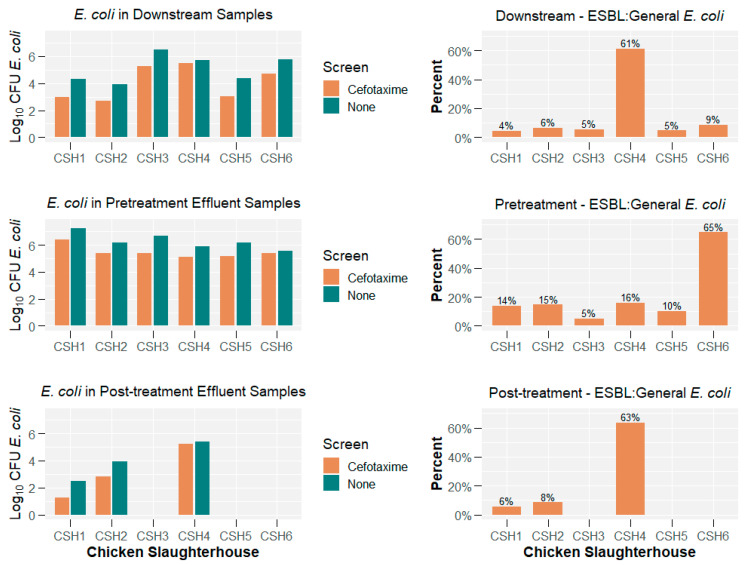
Concentration of ESBL *E. coli* and general *E. coli* across different slaughterhouses and sampling locations.

The Tricycle Protocol generated results on ESBL and general *E. coli* concentrations for six chicken slaughterhouses (effluent, upstream and downstream). Figure 1 displays the log10-transformed *E. coli* concentrations for (1) ESBL *E. coli*, (2) general *E. coli*, and (3) the ratio between ESBL and general *E. coli*.

The median concentrations of ESBL *E. coli* from upstream river samples was 21,244.85 (log10 4.33) CFU/100 mL and from downstream river samples was 24,648.60 (log10 4.39) CFU/100 mL. The median concentrations of ESBL *E. coli* from pre-treatment and post-treatment effluent samples were 229,545.50 (log10 5.36) CFU/100 mL and 709.10 (log10 2.85) CFU/100 mL, respectively. The proportion of ESBL *E. coli* to general *E. coli* in river water samples ranged from 3.5 to 61.3% (mean = 12.2% and median = 6.2%), while effluent samples ranged from 5.0 to 64.7% (mean = 22.4% and median = 13.8%). The proportions varied widely (9 of 12 were between 3.5 and 8.6%); however, in effluent direct from the slaughterhouse, the ratio appeared to increase (5 of 6 were >10.1%).

**Figure 2:**
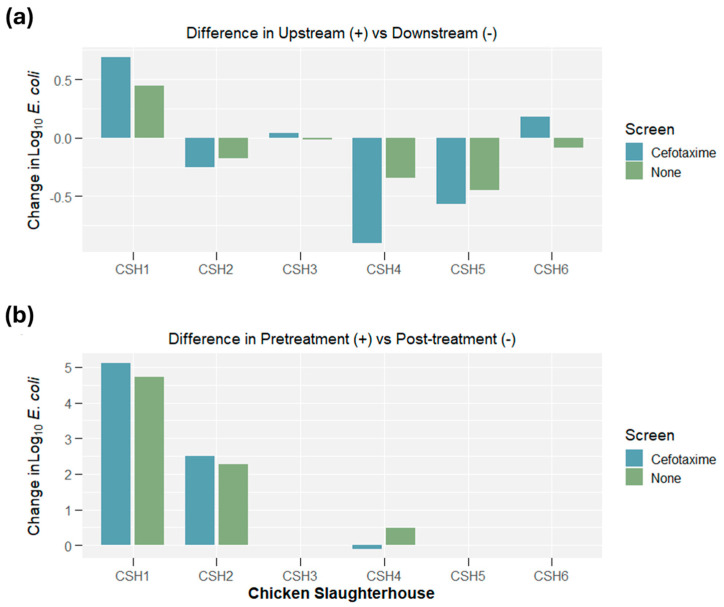
(**a**) Differences in *E. coli* concentrations between upstream and downstream samples and (**b**) pre-treatment and post-treatment samples. ‘+’ denotes that if the concentration was a positive value, there was a higher concentration in ‘Upstream’ or ‘Pre-treatment’ samples.

Comparing the upstream–downstream change in log10 *E. coli* concentration for both ESBL and general *E. coli* (see Figure 2), seven of twelve measurements suggested that the log10 *E. coli* concentration (either general or ESBL) increased downstream compared to upstream (three decreased and two remained the same). Finally, the data suggested that five of the six measurements in log10 *E. coli* concentration between pre-treatment and post-treatment samples had pre-treatment concentrations higher than post-treatment concentrations (one remained the same).

### 2.2. Whole Genome Sequencing: Oxford Nanopore vs. Illumina

The suggested threshold for the minimum total Mbases per sample (200 Mbases set by PulseNet International) was obtained for all but two samples sequenced on the Oxford Nanopore MinION and all samples sequenced on the Illumina MiSeq. For the Nanopore MinION data, the mean read quality post filtering was Q9.9, and per run, contamination levels were below the 5% threshold for all isolates. Finally, mean assembly coverage was 96x and the median number of contigs classified as chromosomal was one. For the Illumina MiSeq data, the mean read quality post filtering was Q36, per isolate, contamination levels were below 5% for all but one isolate (removed for subsequent analyses), the mean assembly coverage was 109x and the median number of contigs classified as chromosomal was 52. Table 1 and Table S1 provide sequencing and assembly statistics.

Final polished isolate assemblies of the long-read data from the Nanopore MinION were compared to polished isolate assemblies from short-read data of the Illumina MiSeq, hybrid assemblies, and to phenotypic properties generated by AST. The concordance between long-read assemblies and hybrid assemblies was consistently higher than the concordance between short-read assemblies and hybrid assemblies for ARG identification (98% vs. 88%, respectively, *n* = 114 ARGs), identifying the genetic component—plasmid or chromosome—hosting the ARG (99% vs. 66%, respectively, *n* = 114 ARGs), identifying virulence factors (96% vs. 90%, respectively, *n* = 209 virulence factors), identifying the genetic component hosting the virulence factor (96% vs. 79%, respectively, *n* = 209 virulence factors), and serotype (100% vs. 98%, respectively, *n* = 42 isolates). See Tables S2–S5 in the Supplementary Material for additional detail. However, when single-nucleotide polymorphism-based (SNP) methods were used, the short-read assemblies performed better (cgMLST and Prokka annotated phylogenetic trees). Phylogenetic trees demonstrated more consistent clustering between short-read assemblies and hybrid assemblies of the same isolate compared to long-read assemblies. Next, the Tricycle Protocol and AST ARG presence/absence results were compared to the long-read assembly results. Table S6 lists the antibiotic tested and positive identification across phenotypic (AST/Tricycle) and genotypic (Nanopore) approaches. The concordance of the long-read assemblies with the AST and Tricycle methods was 91% (143/158 phenotypic opportunities). The AMR classes with concordances below 90% included Phenicol (3/5, 60%), Fluoroquinolones (23/28, 82%), Aminoglycosides (11/13, 85%), and Tetracycline (20/23, 87%) while concordance for B-lactamases was 98% (50/51). Finally, the bioinformatic pipelines built on Galaxy Europe for both long-reads (Nanopore) and short-reads (Illumina) were compared to previously validated ‘non-Galaxy’ pipelines (command line tools available on Github). The outputs and results suggested both pipelines were concordant between the Galaxy and non-Galaxy implementation.

### 2.3. Whole Genome Sequencing: Oxford Nanopore

The sequenced isolates were first evaluated for the presence of ESBL genes. Overall, 20 of 21 (95%) phenotypically confirmed ESBL isolates had an ESBL gene present, while 13 of 21 (62%) of the general isolates (no antibiotic selection) had an ESBL gene present. Among the phenotypically confirmed ESBL isolates, a blaCTX-M gene was present on 19 of 21 (90%) isolates with 1 isolate having only a blaTEM gene and 9 of 21 (43%) having both a blaCTX-M and blaTEM genes present. Of the blaCTX-M genes present in the phenotypically confirmed ESBL isolates (*n* = 21), 14% (3 of 21) were blaCTX-M-1, 14% (3 of 21) were blaCTX-M-15, and 67% (14 of 21) were blaCTX-M-55 (1 was blaCTX-M-27). Among the general isolates, blaTEM was most common (12 of 21; 57%) and blaCTX-M was only present in 4 of 21 (19%) isolates (3 blaCTX-M-55 and 1 blaCTX-M-180). In addition, 19 of 21 (90%) phenotypically confirmed ESBL isolates carried the ESBL gene(s) on a plasmid, while 4 of 21 (19%) carried the ESBL gene(s) on the chromosome (2 isolates harbored ESBL gene(s) on both). For general isolates, 12 of 21 (57%) carried the ESBL gene(s) on a plasmid, while 2 of 21 (10%) carried them on the chromosome (1 isolate harbored ESBL gene(s) on both). See Figure S2 in the Supplementary Material for additional detail.

**Figure 3:**
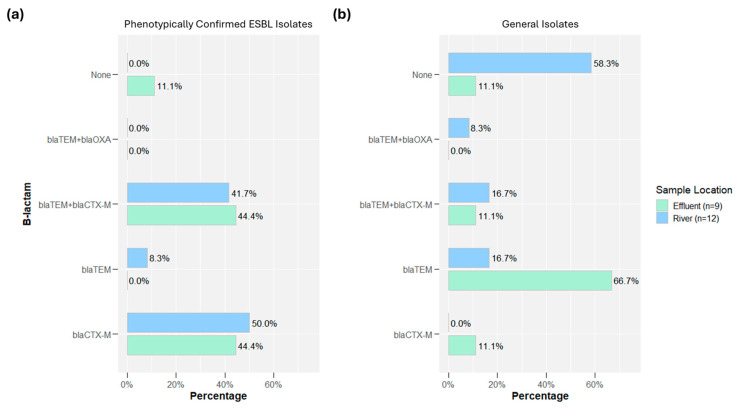
Prevalence of ESBL genes among (**a**) phenotypically confirmed ESBL isolates (*n* = 21) and (**b**) general isolates (*n* = 21). The one blaOXA gene was blaOXA-10.

Figure 3 depicts the distribution of ESBL genes between river samples (*n* = 12, one upstream and one downstream at each chicken slaughterhouse) and effluent samples (*n* = 9, pre-treatment effluent for six slaughterhouses and post treatment of three slaughterhouses that had treatment). Among the phenotypically confirmed ESBL isolates, all isolate genomes contained either blaCTX-M only or blaCTX-M and blaTEM except two isolates (one isolate carried only the blaTEM gene, while the other carried no specific ARG related to the ESBL phenotype). Both river and effluent sample proportions were similar between the two groupings. For the general isolates, the majority of *E. coli* isolated from river samples had no ESBL genes, while the majority of effluent samples had the blaTEM gene. However, the majority of the blaTEM genes were blaTEM-1B/C/D, with one blaTEM-128 gene variant.

**Figure 4:**
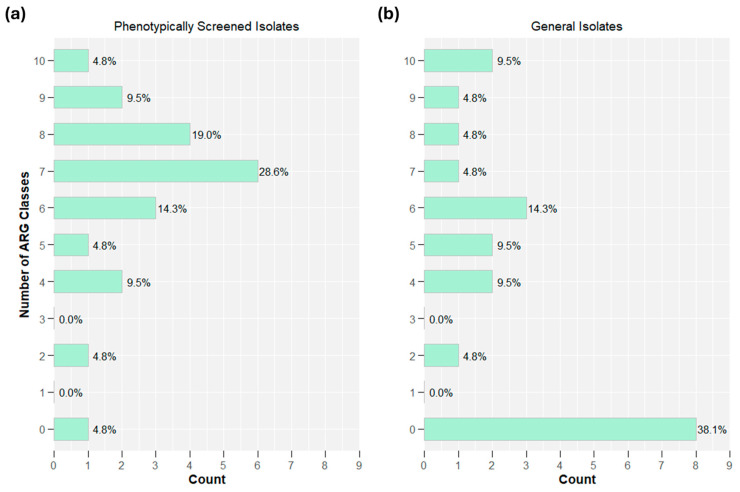
Multi-drug resistance among (**a**) phenotypically confirmed ESBL isolates (*n* = 21) and (**b**) general isolates (*n* = 21).

Figure 4 depicts the percentage of isolates that carried ARGs associated with unique ARG drug classes for both phenotypically confirmed ESBL isolates and general isolates. Among the phenotypically confirmed ESBL isolates, only 1 isolate was not genotypically multidrug resistant (defined as hosting genes from two or more drug classes), while 16 of 21 (76%) isolates carried ARGs from ≥6 drug classes. Among general isolates, 8 of 21 (38%) isolates carried no ARGs, while 8 of 21 (38%) general isolates carried ARGs for ≥6 drug classes. Figure S3 disaggregates the samples by sampling location (river vs. effluent). For phenotypically confirmed ESBL isolates, there was little difference between the river or effluent samples, but for the general isolates, the majority of river isolates carried no ARGs, while 89% of effluent samples carried ARGs for at least four drug classes.

**Figure 5:**
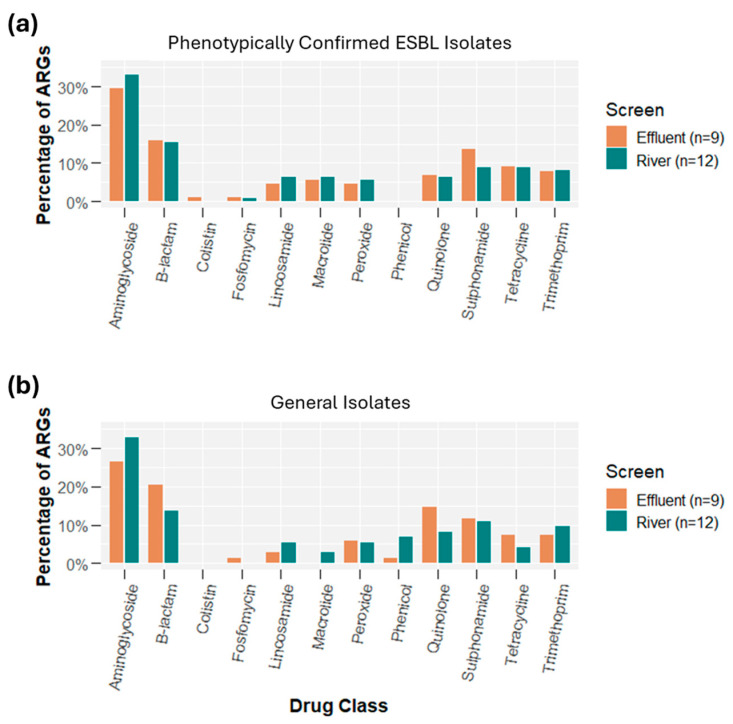
Prevalence of ARGs by drug class disaggregated by (**a**) phenotypically confirmed ESBL isolates and (**b**) general isolates and by sampling location.

Among all isolates, there were 353 total ARGs, 298 (84%) carried by plasmids and 55 (16%) carried on chromosomes (see Figure S3). Of the 353 total ARGs, there were 48 unique ARGs and associated variants (as denoted by ResFinder4.1). Among the phenotypically confirmed ESBL isolates, there were 214 total ARGs, 171 (80%) carried by plasmids and 43 (20%) carried on chromosomes. For general isolates, there were 139 total ARGs, 127 (91%) carried by plasmids and 12 (9%) carried on chromosomes. Figure 5 and Figure S4 present the abundance of all ARGs by phenotypically confirmed ESBL versus general isolates and by sampling location (effluent vs. river). Of particular note is that all Quinolone resistance genes were found on plasmids. In addition, all Phenicol resistance gene were found in the isolates not phenotypically confirmed for ESBL (i.e., general isolates). A higher proportion of effluent isolates had B-lactam resistance (ESBL), confirming Figure 3; however, river samples had a higher proportion of Aminoglycoside, Phenicol, Macrolide, and Lincosamide resistance genes. In addition to B-lactam resistance genes, effluent samples also had a higher proportion of Quinolone, Sulphonamide, and Tetracycline resistance genes.

Figures S5–S7 depict the distribution of pathotypes identified via virulence factors among both groups of isolates. For both groups, intestinal pathogenic *E. coli* (IPEC) was only identified once, while extraintestinal pathogenic *E. coli* (ExPEC) had a higher prevalence. However, phenotypically confirmed ESBL isolates were classified as ExPEC more often than general isolates (62% vs. 43%, respectively). Overall, of the general isolates, 11 of 21 (52%) were non-pathogenic, while among the phenotypically confirmed ESBL isolates, only 7 of 21 (33%) were non-pathogenic.

Finally, to evaluate relatedness between isolates, phylogenetic trees were constructed based on shared core genes (genes present in ≥95% of isolates). Figures S8 and S9 depict the phylogenetic trees for the phenotypically confirmed ESBL isolates and the general isolates, respectively. The sampling location, pathotype, and presence of ARGs and plasmids were compared to clusters within the phylogenetic tree. However, no significant trends were identified given the metadata evaluated. Further details are provided in the Supplementary Material.

### 2.4. Cost Analysis

The cost of Nanopore sequencing will depend on the intended use within the surveillance system, the market cost of the reagents, and the ease of access to reagents. Table S7 presents the costs of reagents (as of 2019) used in this isolate-based portion of the pilot study separated into capital costs, reagent costs, and data management costs. No personnel costs are included in this initial table. Costs per sample were estimated but did not include reagents used for training sessions. Table 2 presents the total cost per sample, which includes capital costs (e.g., laptop, MinIT device, hard drives) and the cost per sample excluding capital costs to provide information for the extended use of this approach. Finally, we provide a cost estimate when optimal lab processing conditions are met (eight barcoded samples per run; excluding capital costs) as well as the optimal cost for isolation using the Tricycle Protocol.

## 3. Discussion

The first question evaluated by this pilot study was as follows: what types and concentrations of *E. coli* are present in the effluent of chicken slaughterhouses and the receiving rivers? The results suggest that while both general and ESBL *E. coli* concentrations were higher in effluent, these values were within the range of values reported globally for rivers [16,17]. However, for recreational water bodies (e.g., rivers in which humans swim or play), EPA recommendations are for a geometric mean of 126 CFU/100 mL for *E. coli* [18]. The proportion of ESBL *E. coli* to general *E. coli* in this pilot study demonstrated that pre-treated effluent ratios were twice as high as river ratios (median proportions: 14.2% vs. 6.2%, respectively). Previous data from Wang and colleagues evaluated *E. coli* levels across types of water sources from 10 countries, with open drain and flood water having the highest levels of *E. coli* concentrations (range of means 4.38 to 7.99 log10 values) [17]. However, even our limited data support the extensive body of literature on the effectiveness of treatment technologies on AMR control [19,20,21,22]. The two slaughterhouses which had multi-stage, fully operational effluent treatment demonstrated reductions in *E. coli* and the ratios of ESBL to general *E. coli* (general *E. coli* 6.72 log10 to 3.22 log10 and a 7.2% ratio reduction). Identifying cost-effective treatment methods adapted to local approaches can provide critical control mechanisms for the spread of AMR.

Isolate characterization suggested that among the general isolates (those with no specific AMR selection), 6 of 9 effluent isolates carried blaTEM (one had no ESBL genes), while 7 of 12 river isolates had no ESBL genes (Figure 3). Only 6 of 21 general isolates carried an ESBL gene that was not blaTEM 1B/C/D. Among the phenotypically confirmed isolates, 14 of 21 blaCTX-M genes isolated were blaCTX-M-55 (similar proportions across effluent and rivers). Evaluating all drug classes among general isolates, in rivers, the most prevalent ARGs included genes conferring resistance to aminoglycosides (33%), beta-lactams (14%), and sulphonamides (11%), while in effluent, genes conferring resistance to aminoglycosides (26%), beta-lactams (20%), and quinolones (14%) were most prevalent. According to the FAO and DGLAHS MoA internal survey data on antimicrobial usage in broiler poultry in 2020, enrofloxacin and amoxicillin–colistin were among the most extensively used by poultry producers in Indonesia [23]. However, colistin use has decreased significantly since 2017 [24]. The intensive use of antibiotics in 2020 was aimed to prevent infectious diseases in broilers. This could result in *E. coli* resistance to antibiotic classes found in slaughterhouse effluents. When isolates were phenotypically screened for ESBLs, the results of sequencing suggested that the prevalence for ARGs in isolates from rivers and effluent was expectantly similar. However, interestingly, for these screened ESBL isolates, 20 of 21 isolates were MDR. Among the general isolates, 13 of 21 were MDR. Previous work in Portugal reported that 72% of *E. coli* isolates from river water samples exhibited AMR, while 28% were resistant to six or more drug classes, underscoring the environmental dissemination of MDR strains linked to human and animal activities [25]. Additionally, a study in Nigeria found a high prevalence (45%) of MDR *E. coli* in cloacal swabs from poultry, highlighting poultry farms as significant reservoirs for antibiotic resistance dissemination [26].

For gene mobility, whole genome sequencing and gene annotation demonstrated that ARGs were typically carried by plasmids. Previous studies have shown that ESBL-producing *E. coli* from poultry environments frequently harbor ARGs on mobile genetic elements like plasmids, facilitating their dissemination in surrounding water systems and effluents [27,28]. Finally, the pathotype of isolates was evaluated via the virulence factors present in the genome assemblies. Only two isolate genomes contained virulence genes associated with IPECs (both isolated from river samples). However, the majority of isolate genomes contained virulence genes associated with ExPECs (22 of 42; see Figure S4). Consistent with our findings, research on *E. coli* from agricultural and riverine environments has demonstrated a higher prevalence of ExPEC-associated virulence genes compared to IPEC-associated genes, suggesting a risk of extra-intestinal infections linked to these environments [29,30].

The second question evaluated by this pilot study was as follows: can the Oxford Nanopore MinION sequencing technology provide valid and valuable data for AMR monitoring systems currently deploying the Tricycle Protocol? To evaluate the validity of data generated by the Nanopore MinION, outputs were compared to two ‘gold standard’ methods, namely, hybrid short-long read assemblies (following the PulseNet International protocol for Illumina MiSeq sequencing) and AST. The comparison between the long-read assemblies (i.e., MinION) and hybrid assemblies enabled evaluations of sequencing outputs and bioinformatic pipelines (using open-source, non-command line tools). Several recent studies have reviewed the accuracy of short-read, long-read, and hybrid assemblies, identifying pro and cons to both approaches [31,32,33]. Recently, Foster-Nyarko and colleagues evaluated Nanopore-only performance, suggesting current methods (used in this study) are robust for key isolate characteristics; however, due to the high error rate, cluster analysis in phylogenetic trees is still limited. This was confirmed in this pilot study. Polished assemblies from the hybrid and long-read pipelines demonstrated robust concordance for ARGs (98%), virulence factors (96%), and serotypes (100%). However, the cgMLST and phylogenetic trees (using cgMLST) struggled to align. Interestingly, Foster-Nyarko and colleagues suggest that an SNP limit of 50 may be necessary to rule out a long-read-only assembly from a cluster within a tree. This suggests that improved error rates or assembly correction may be needed prior to using both long-read and short-read assemblies within the same phylogenetic tree. Lastly, the ARGs identified using the long-read assemblies achieved a concordance of 91% with the phenotypic AST results. These results provide initial evidence that the Nanopore MinION can generate comparable results to gold standard genetic and phenotypic methods.

The comparison between the long-read and short-read bioinformatic pipelines used here is important in two ways. First, the short-read pipeline has been available since 2013 and validated [34], where the long-read pipeline is still being tested by various stakeholders (personal communication). Second, a key aim in this pilot study was to utilize free, open-source, and easily deployable bioinformatics tools, and therefore, the adaptation of both pipelines from command line-based tools to the browser-based Galaxy Europe tools and gene identification and annotation tools on the Center for Genomic Epidemiology’s website needed to be validated. Adaptation comparisons suggested that differences in outputs were negligible between bioinformatic platforms used.

The Nanopore MinION can provide flexibility in national AMR surveillance systems but also comes with specific limitations. If certain efficiencies are obtained by the laboratory, per sample costs can be around USD $80 or lower excluding capital costs. However, capital costs are minimal, enabling more equitable access supported by the simplified library preparation (reducing lab time and the need for extensive training). Comparatively, Illumina sequencing equipment is not mobile and can cost at least USD $100–225 per sample depending on operations and batching. While the MinION device is extremely portable, the reagents are less portable and often have a short shelf life. For example, both the Rapid Barcoding kit and flow cells must be stored at 2–8 °C and have a 90-day warranty (Oxford Nanopore Technologies, United Kingdom). However, experience suggests flow cells can last up to a year in proper storage while new kits reduce the need for cold storage. To normalize fluctuations in reagent needs in a given region, a centralized distribution channel needs to be established to help increase access and decrease the chance of expired reagents. However, supply chains and import processes can inhibit efficient and sustainable use if not addressed appropriately. Finally, the frequency of sampling monitoring points will be site specific. The World Health Organization (WHO) has recommended that for the Tricycle Method, samples from an individual site should be taken monthly [9]. If monetarily feasible, all isolates should be sequenced, but an agreed upon subsample can also be used. This should be selected based on the monitoring points in a region and environmental factors, as discussed in the WHO Tricycle Method handbook. If these operational hurdles can be overcome, the additional information provided by sequencing, as demonstrated by this pilot study, can be a valuable information asset to current national AMR surveillance systems.

Limitations in this pilot study included variation in slaughterhouse characteristics, sample size and cross-sectional sampling at different time points, seasonality, and sequencing disruptions. As the study resources and timeline were limited, only six slaughterhouses were selected for evaluation via a cross-sectional study design. However, cross-sectional sampling was conducted over several months (not all on one day). This can contribute to variation in results due to rain events and general seasonality, especially for the river sampling. However, the primary focus of this pilot study was on testing sampling, laboratory, and bioinformatic protocols. In addition, strict sampling criteria were used to improve the level of standardization in sampling environments (see the Supplementary Material for details). Finally, for two isolates, Nanopore sequencing had been initiated, but was then disrupted due to power or internal processing errors. When this occurred, the system was restarted following the manufacturer’s instructions.

## 4. Materials and Methods

### 4.1. Study Site and Sample Collection

The pilot study was conducted in the Greater Jakarta area in Indonesia where a total of six chicken slaughterhouses were sampled. The number of chickens slaughtered per day ranged from 700 to 30,000 per day, with four locations below 4000 and the remaining two locations above 24,000. Three slaughterhouses operated during the day and three during the night, while three of the six utilized some type of effluent treatment. All slaughterhouse effluent was discharged into adjacent rivers. Samples were collected during operational hours and more than 12 h after any significant rain event (>2 mm over five hours).

**Figure 6:**
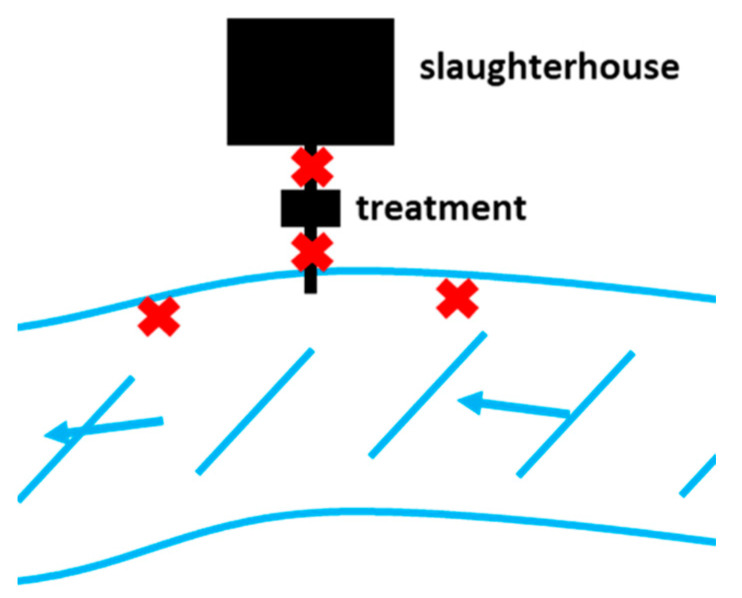
Diagram of sampling points. Blue denotes the river, red ‘X’ denotes sampling points.

Figure 6 depicts the sampling locations at each chicken slaughterhouse (*n* = 6) for the pilot study. At each slaughterhouse, three or four sampling locations were identified. If the slaughterhouse had any type of effluent treatment (*n* = 3), samples were collected before and after treatment (but before entering the river). At all slaughterhouses, river samples were collected both 10 m upstream and downstream from the discharge point.

For each sampling point, 1000 mL of sample (river water or effluent) was collected utilizing a 300 mL collection beaker and acquiring four samples spaced 1–2 min apart and homogenizing the sample via light physical shaking for 30 s. Next, the sample was immediately allocated into two sterile glass bottles, a 600 mL bottle and a 250 mL bottle, for direct DNA extraction for metagenomic sequencing and the Tricycle Protocol, respectively. The samples were placed in a cooler and transported back to the Quality Control and Animal Product Certification Laboratory (Balai Pengujian Mutu dan Sertifikasi Produk Hewan; BPMSPH) in Bogor, West Java. In addition, a short survey was used to collect information about the chicken slaughterhouse, the local environment, and the sampling conditions. Additional details of the sampling approach are presented in the Supplementary Material.

### 4.2. Tricycle Protocol and Antimicrobial Susceptibility Testing (AST)

**Figure 7:**
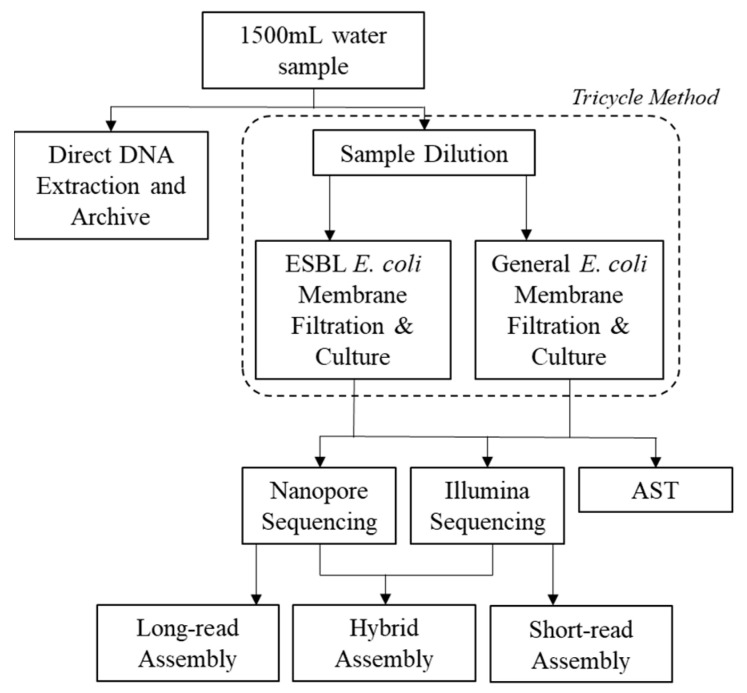
Depiction of the study workflow including Tricycle Protocol for ESBL and general *E. coli* enumeration, sequencing, and antimicrobial susceptibility testing (AST).

On arrival at the laboratory, the 250 mL sample was once again homogenized via light physical shaking for 30 s. The enumeration, isolation, and identification of total and ESBL *E. coli* from effluent and river water samples (including ESBL *E. coli* confirmatory tests) were conducted following the membrane filtration version of the Tricycle Protocol [8] (see the full workflow in Figure 7 and a detailed protocol in the Supplementary Material). Briefly, membrane filtration was conducted on a diluted sample (10-fold using PBS) and transferred to a plate with TBX medium for general *E. coli* enumeration (a TBX–cefotaxime plate was used for the enumeration of presumptive ESBL *E. coli*). Plates were incubated at 37° C for 24 h. After the enumeration of general *E. coli* and presumptive ESBL *E. coli*, 10 general *E. coli* colonies and 10 ESBL *E. coli* colonies were picked from each plate and streaked onto either a MacConkey agar plate (general *E. coli*) or a MacConkey–cefotaxime plate. Plates were incubated at 37 °C for 24 h. Finally, colonies were picked and transferred to a nutrient agar plate and incubated at 37 °C for 12 h.

To facilitate the identification of *E. coli*, the Sulfide Indole Motility (SIM) test, methyl red and Voges–Proskauer (MRVP) tests, and citrate tests were conducted (see the Supplementary Material for details). Finally, the double-disk method (DDT) method was used to confirm ESBL *E. coli* colonies using cefotaxime, ceftazidime, a combination disk of cefotaxime and clavulanic acid, and a combination disk of ceftazidime and clavulanic acid. Bacterial concentrations were then calculated based on the initial enumeration, total volume plated, number of picked colonies, and number of confirmed colonies (we use the term ‘phenotypically confirmed ESBL’ from here on to refer to the presumptive ESBL *E. coli*). Finally, isolates were preserved in glycerol mixed with culture media and stored at −80 °C if further testing was not conducted immediately.

Antimicrobial susceptibility testing (AST) for each isolate was conducted using the EU Surveillance Salmonella/*E. coli* (EUVSEC) plate. The Sensititre plate contained 14 different antibiotics (see the Supplementary Material for further details). Following the manufacturer’s protocol, the minimum inhibitory concentrations (MICs) for each antibiotic were determined for all isolates. In this study, we define multi-drug resistance (MDR) for phenotypic testing as any isolate that demonstrates an MIC classified as resistant by the Clinical and Laboratory Standards Institute (CLSI) and European Committee on Antimicrobial Susceptibility Testing (EUCAST) breakpoints for at least two antimicrobial agents, while for genotypic sequencing, this includes any isolate that carries two or more ARGs from different antimicrobial resistance drug classes.

### 4.3. Whole Genome Sequencing: Oxford Nanopore and Illumina

Whole genome sequencing on the Oxford Nanopore MinION Mk1B (Oxford Nanopore Technologies, Oxford, UK) was conducted at the BPMSPH laboratory. Briefly, the DNA extraction of the *E. coli* isolates was conducted using the Qiagen DNeasy Blood and Tissue extraction kit (Hilden, Germany) following the manufacturer’s protocol. DNA quality checks were conducted using the NanoDrop 2000 (ThermoFisher, Waltham, MA, USA) (no Qubit was available). Next, library preparation was conducted using the Rapid Barcoding Sequencing (SQK-RBK004) kit following the manufacturer’s protocol. Initial sequencing runs included only two barcoded isolates per run but increased to four by the last three slaughterhouses (see the Supplementary Material for more information). MinION R9.4 flow cells were used for sequencing.

Whole genome sequencing on the Illumina MiSeq (Illumina, Inc., San Diego, CA, USA) was conducted on a subset of DNA extracts from the *E. coli* isolates at the Disease Investigation Center (DIC) Wates in Yogyakarta, Indonesia. The pure DNA of *E. coli* isolates were transferred on ice to the lab. The PulseNet International protocol for library preparation and sequencing for the Illumina MiSeq was used (Centers for Disease Control and Prevention, 2019b [35]). The Nextera DNA XT kit (Illumina, Inc.) was used for library preparation, and sequencing was conducted using the v2 kit with 500 cycles for a read length of 2 × 250 bp.

### 4.4. Quality Control

Several methods were used to provide quality control and contamination checks throughout the pilot study based on available resources. These included field blanks, deionized water transferred from a storage container to a collection bottle in the field and included in the direct DNA extractions. In addition, deionized water extraction blanks were used for the isolate DNA extractions (post culture). Quality control strains for the culture-based isolation included *E. coli* 10,455 NCSU and Klebsiella pneumoniae ATCC 70 for isolation, identification, and confirmatory tests of ESBL *E. coli*. *E. coli* ATCC 25,922 was used as the control strain for the isolation and identification of general *E. coli*. Finally, during the initial DNA sequencing of barcoded isolates, the lambda control provided in the Nanopore Rapid Barcoding Ligation Kit was included as one of the barcoded samples. The results were validated following the manufacturer’s guidelines.

### 4.5. Bioinformatic Analyses

For the Nanopore sequencing data, the MinIT device was used to conduct basecalling (using default settings of Guppy v2.2.3) and fast5 and fastq data were transferred to cloud storage and duplicated on an external hard drive. All fastq files were automatically uploaded to Epi2Me (https://epi2me.nanoporetech.com/, accessed on 1 June 2020) for initial contamination and quality control checks. Centrifuge and the NCBI reference database was used to classify reads [36]. Any sequenced isolate with >5% reads classified as something other than *Escherichia* at the genus level was deemed contaminated.

The fastq files were uploaded to Galaxy Europe [37] for assembly and evaluation. The bioinformatic protocol nanopore Workflow v0.4.4 developed by Katz and Kapsak 2020 [38] was adapted for use on Galaxy Europe (see Figure S1 for a pipeline depiction). Briefly, reads were filtered using filtlong v0.2.0 [39] with default parameters, and the highest quality reads totaling 600 Mbases were selected for downstream analysis. Quality check (QC) reports were generated with NanoPlot v1.28.2 [40]. The initial assembly was conducted by Flye v2.3.7 (default settings, estimated genome size 5 m) [41], while the filtered fastq files were converted to fasta files (FASTQ to FASTA converter v1.1.5). Next, minimap2 v2.17 [42] was used to align reads to the draft assembly from Flye, and Racon v1.3.1.1 [43] was used for contig consensus correction. This correction process was repeated four times as recommended by the medaka documentation [44]. Finally, medaka v1.0.1 (Oxford Nanopore Technologies Ltd., Oxford, UK) was used as a final consensus correction step before evaluation. PlasFlow v1.0 was used to differentiate between chromosome and plasmid contigs [45]. Finally, the adapted long-read isolate pipeline on Galaxy Europe was compared with the originally developed long-read isolate pipeline available on Github (https://github.com/kapsakcj/nanoporeWorkflow, accessed on 8 December 2020) by running the same test isolate fastq file on both pipelines. This was also conducted for the adapted short-read pipeline available on Github (https://github.com/lskatz/SneakerNet, accessed on 8 December 2020) using a test short-read fastq file.

For Illumina, the protocol developed by Katz 2020 [34,46] was adapted for use on Galaxy Europe. Briefly, Trimmomatic v0.38 was used to filter reads below Q20 [47]. QC reports were generated by FastQC v0.72 and MultiQC v1.9 visualization [48] from raw paired-end reads (independent of R1/R2). Next, Kraken v2.0 [49] was run on the merged paired-end reads (default parameters and standard database). Any sequenced isolate with >5% reads classified as something other than *Escherichia* at the genus level was deemed contaminated. Finally, Shovill v1.1.0 [50] with the skesa assembler [51] was used for filtering, assembly, and polishing followed by Prokka v1.14.6 (using Prodigal for gene prediction [52]) for gene prediction and annotation [53].

For hybrid assemblies, paired-end reads from Illumina sequencing were filtered via Trimmomatic v0.38, while single long-reads from Nanopore sequencing were filtered via filtlong v0.2.0. SPAdes via Unicycler v0.4.8 [54] was used with default parameters to generate hybrid assemblies, and PlasFlow v1.0 was used to differentiate contigs associated with the chromosome and potential plasmids.

Final assemblies for all isolates (Nanopore, Illumina, and hybrid assembles) were then uploaded to the following tools on the Center for Genomic Epidemiology website (http://www.genomicepidemiology.org/services/, accessed on 10 May 2021), KmerFinder v3.2 (bacteria organism) [55], ResFinder v4.1 [56] (Escherichia coli*, assembled genome/contigs, 98% identification threshold, 98% minimum length), VirulenceFinder 2.0 [57] (*Escherichia coli*, 98% identification threshold, 98% minimum length, assembled or draft genome/contigs), and PlasmidFinder v2.1 [58] (*Enterobacteriaceae*, 98% identification threshold, 98% minimum length, assembled or draft genome/contigs). Isolates were classified by pathotype using the virulence genes present, following the protocols from Franz et al. 2015 [59] and Sarowska et al. 2019 [60]. Finally, to generate phylogenetic trees, Roary v3.13.0 [61] (95% blastp identity, 95% of genes for core classification), RAxML v8.2.4 [62] (default), and iTOL v4 [63] were used. Phylogenetic trees were used to visualize isolate clusters and evaluate similarities between sequencing platforms and assembly approaches (Nanopore only, Illumina only, and hybrid).

### 4.6. Analyses

For the Tricycle Protocol data, concentrations and proportions of *E. coli* were estimated. Next, to compare AST-identified phenotypic properties with the associated genes, concordances were calculated for a subset of the isolates (*n* = 21) for the Nanopore MinION. To compare the genotypic validity of the Nanopore MinION, results from the identification of ARGs, virulence factors, serotypes, and cgMLST were compared to the ‘gold standard’ hybrid assemblies. In addition, to further evaluate gene annotation and cluster capabilities (e.g., for outbreak investigations), integrated phylogenetic trees were estimated using the method described above for all matched isolates (*n* = 21; Nanopore only, Illumina only, and hybrid). Trees were then compared for similar structure and clusters. Finally, costs for the pilot study were used to evaluate a per sample cost, both including and excluding capital costs (i.e., laptop, MinIT device, external hard drive, etc.; see the Supplementary Material for details).

## 5. Conclusions

This pilot study aimed to evaluate (1) *E. coli* from chicken slaughterhouses and (2) the feasibility of using the Oxford Nanopore MinION in national AMR surveillance systems. The results suggest that chicken slaughterhouse effluent has higher concentrations of *E. coli* and a higher abundance and diversity of ARGs compared to receiving rivers. However, locally adapted treatment may be able to provide a critical control mechanism when appropriate. The Nanopore MinION produced similar outputs to hybrid assemblies (via Illumina MiSeq) and AST. Maintaining standardization and rigor while increasing access and options is critical for locally optimizing the AMR surveillance of critical control points in one-health systems.

## Figures and Tables

**Table 1:** Sequencing and assembly statistics for Oxford Nanopore (*n* = 42) and Illumina (*n* = 19).

Results per Run	Oxford Nanopore MinION Mean (Range)	Illumina MiSeq Mean (Range)
QC		
Total Mbases	479.6 (54.5–1400)	-
Total Mbases (post QC)	391.4 (59.4–600 ^)	273 (110–404)
Total reads (post QC)	82,432 (14,790–167,621)	1,215,360 (555,750–1,606,430)
Read quality (post QC)	Q9.91 (8.8–11.2)	Q36 (35.28–36.09)
Read length (post QC)	4810 bp (2691–10,511)	236 bp (215–247)
N50 (post QC)	6819 bp (4031–11,748)	-
Contamination *		
Per run (%)	Classified reads: (0.97–3.55) Total reads: (1.10–3.75)	-
Per isolate (%)	Classified reads: (0.22–5.79) Total reads: (0.25–6.25)	Classified reads: (0.36–4.87) ^ Total reads: (0.58–5.84)
Assembly **		
Estimated coverage; mean (median)	96x (84x)	109x (113x)
Total chromosomal contigs; mean (median)	3.5 (1)	54.7 (51.5)
Total plasmid contigs; mean (median)	5.4 (4)	39.1 (38.5)

* Contamination is defined as any read not classified as Escherichia (genus level). ** Assembly after polishing and consensus. ^ The QC step took the top 600Mbases for assembly if there were more total Mbases than 600. ^ Does not include contaminated isolate that was removed from subsequent analyses.

**Table 2:** Costs and capabilities of approaches and technologies for isolate analyses.

	Tricycle Protocol	MinION Sequencing	MiSeq Sequencing	Hybrid Approach	Senstitire AST
Pilot Study Cost per Sample	USD $10	USD $292	USD $225	USD $517	USD $20
Projected Cost per Sample at Scale *	USD $5	USD $70–100	USD $80–150	USD $150–250	USD $12
					
ESBL *E. coli* Concentration	**X**				
Total *E. coli* Concentration	**X**				
ESBL to Total *E. coli* Ratio	**X**				
ARG Identification		**X**	x	**X**	**X**
ARG Location		**X**	x	**X**	
Virulence Factor Identification		**X**	x	**X**	
Virulence Factor Location		**X**	x	**X**	
Serotype		**X**	x	**X**	
Phylogenetic Relatedness		x	**X**	**X**	

Smaller ‘x’ denotes where a method was assessed to be less capable in our analysis. * Estimates from local suppliers in Indonesia. A ‘sample’ for the sequencing and AST methods refer to an isolate.

## Data Availability

All data are provided in the Supplementary Materials, except the raw sequencing files. These are retained by the Government of Indonesia and may be provided upon request.

## Supplementary Materials

The following supporting information can be downloaded at: https://www.mdpi.com/article/10.3390/antibiotics14070624/s1, Figure S1: Depiction of bioinformatic pipelines; Table S1: Results on QC, contamination, and assembly; Table S2: Number of ARGs identified by Nanopore, Illumina, and hybrid assemblies and concordances; Table S3: VFs identified by Nanopore, Illumina, and hybrid assemblies and concordances; Table S4: Comparison of data sets generated by Nanopore, Illumina, and hybrid assemblies with SeroType; Table S5: Comparison of data sets generated by Nanopore, Illumina, and Hybrid Assemblies with cgMLST; Table S6: Results of the WGS/Nanopore/AST comparison; Figure S2: Overall distribution of ESBL genes among all samples; Figure S3: Drug resistance among isolates disaggregated by sample location; Figure S4: Total ARGs on all isolates; Figure S5: Prevalence of pathotype among all isolates; Figure S6: Prevalence of pathotype among phenotypically screened ESBL isolates; Figure S7: Prevalence of pathotype among general isolates; Figure S8: Phylogenetic tree for phenotypically screened ESBL isolates; Figure S9: Phylogenetic tree for general isolates; Table S7: Costs of reagents used in this isolate-based portion of the pilot study.

## References

[1] Berendonk T.U., Manaia C.M., Merlin C., Fatta-Kassinos D., Cytryn E., Walsh F., Burgmann H., Sorum H., Norstrom M., Pons M.-N. (2015). Tackling antibiotic resistance: The environmental framework. Nat. Rev. Microbiol..

[2] Naghavi M., Vollset S.E., Ikuta K.S., Swetschinski L.R., Gray A.P., Wool E.E., Aguilar G.R., Mestrovic T., Smith G., Han C. (2024). Global burden of bacterial antimicrobial resistance 1990–2021: A systematic analysis with forecasts to 2050. Lancet.

[3] McEwen S.A., Collignon P. (2017). Antimicrobial resistance: A One Health perspective. Microbiol. Spectr..

[4] Goulas A., Belhadi D., Descamps A., Andremont A., Benoit P., Courtois S., Dagot C., Grall N., Makowski D., Nazaret S. (2020). How effective are strategies to control the dissemination of antibiotic resistance in the environment? A systematic review. Environ. Evid..

[5] Keenum I., Liguori K., Calarco J., Davis B.C., Milligan E., Harwood V.J., Pruden A. (2022). A framework for standardized qPCR-targets and protocols for quantifying antibiotic resistance in surface water, recycled water and wastewater. Crit. Rev. Environ. Sci. Technol..

[6] Liguori K., Keenum I., Davis B.C., Calarco J., Milligan E., Harwood V.J., Pruden A. (2022). Antimicrobial Resistance Monitoring of Water Environments: A Framework for Standardized Methods and Quality Control. Environ. Sci. Technol..

[7] Sano D., Wester A.L., Schmitt H., Amarasiri M., Kirby A., Medlicott K., de Roda Husman A.M. (2020). Updated research agenda for water, sanitation and antimicrobial resistance. J. Water Health.

[8] World Health Organization (2016). Global Tricycle Surveillance: ESBL E. coli.

[9] World Health Organization (2021). WHO Integrated Global Surveillance on ESBL-Producing E. coli Using a “One Health” Approach: Implementation and Opportunities.

[10] Nadon C., Van Walle I., Gerner-Smidt P., Campos J., Chinen I., Concepcion-Acevedo J., Gilpin B., Smith A.M., Kam K.M., Perez E. (2017). PulseNet International: Vision for the implementation of whole genome sequencing (WGS) for global food-borne disease surveillance. Eurosurveillance.

[11] Centers for Disease Control and Prevention (2019). Antibiotic Resistance Threats in the United States.

[12] Day M.J., Hopkins K.L., Wareham D.W., Toleman M.A., Elviss N., Randall L., Teale C., Cleary P., Wiuff C., Doumith M. (2019). Extended-spectrum β-lactamase-producing *Escherichia coli* in human-derived and foodchain-derived samples from England, Wales, and Scotland: An epidemiological surveillance and typing study. Lancet Infect. Dis..

[13] Blaak H., Lynch G., Italiaander R., Hamidjaja R.A., Schets F.M., De Husman A.M.R. (2015). Multidrug-resistant and extended spectrum beta-lactamase-producing *Escherichia coli* in dutch surface water and wastewater. PLoS ONE.

[14] Jorgensen S.B., Soraas A.V., Arnesen L.S., Leegaard T.M., Sundsfjord A., Jenum P.A. (2017). A comparison of extended spectrum β-lactamase producing *Escherichia coli* from clinical, recreational water and wastewater samples associated in time and location. PLoS ONE.

[15] Fuhrmeister E.R., Voth-Gaeddert L.E., Metilda A., Tai A., Batorsky R.E., Veeraraghavan B., Ward H.D., Kang G., Pickering A.J. (2021). Surveillance of potential pathogens and antibiotic resistance in wastewater and surface water from Boston, USA and Vellore, India using long-read metagenomic sequencing. medRxiv.

[16] Berendes D.M., de Mondesert L., Kirby A.E., Yakubu H., Adomako L., Michiel J., Raj S., Robb K., Wang Y., Doe B. (2020). Variation in *E. coli* concentrations in open drains across neighborhoods in Accra, Ghana: The influence of onsite sanitation coverage and interconnectedness of urban environments. Int. J. Hyg. Environ. Health.

[17] Wang Y., Mairinger W., Raj S.J., Yakubu H., Siesel C., Green J., Durry S., Joseph G., Rahman M., Amin N. (2022). Quantitative assessment of exposure to fecal contamination in urban environment across nine cities in low-income and lower-middle-income countries and a city in the United States. Sci. Total Environ..

[18] US Environmental Protection Agency (2012). Recreational Water Quality Criteria 820-F-12-058.

[19] Nguyen A.Q., Vu H.P., Nguyen L.N., Wang Q., Djordjevic S.P., Donner E., Yin H., Nghiem L.D. (2021). Monitoring antibiotic resistance genes in wastewater treatment: Current strategies and future challenges. Sci. Total Environ..

[20] Zhu T.-T., Su Z.-X., Lai W.-X., Zhang Y.-B., Liu Y.-W. (2021). Insights into the fate and removal of antibiotics and antibiotic resistance genes using biological wastewater treatment technology. Sci. Total Environ..

[21] Uluseker C., Kaster K.M., Thorsen K., Basiry D., Shobana S., Jain M., Kumar G., Kommedal R., Pala-Ozkok I. (2021). A Review on Occurrence and Spread of Antibiotic Resistance in Wastewaters and in Wastewater Treatment Plants: Mechanisms and Perspectives. Front. Microbiol..

[22] Wang J., Chen X. (2022). Removal of antibiotic resistance genes (ARGs) in various wastewater treatment processes: An overview. Crit. Rev. Environ. Sci. Technol..

[23] Anwar Sani R., Sunandar S., Rachmawati A., Pertela G., Susanti O., Rahayu K.P., Allamanda P., Suandy I., Nurbiyanti N., Jahja E.J. (2024). Antimicrobial Usage and Antimicrobial Resistance in Commensal *Escherichia coli* from Broiler Farms: A Farm-Level Analysis in West Java, Indonesia. Antibiotics.

[24] FAO in Indonesia (2019). Preserving Critically Important Antibiotics for Humans, by Banning Their Use in Animals.

[25] Bessa L.J., Barbosa-Vasconcelos A., Mendes Â., Vaz-Pires P., Martins da Costa P. (2014). High prevalence of multidrug-resistant *Escherichia coli* and *Enterococcus* spp. in river water, upstream and downstream of a wastewater treatment plant. J. Water Health.

[26] Agusi E.R., Kabantiyok D., Mkpuma N., Atai R.B., Okongwu-Ejike C., Bakare E.L., Budaye J., Sule K.G., Rindaps R.J., James G.K. (2024). Prevalence of multidrug-resistant *Escherichia coli* isolates and virulence gene expression in poultry farms in Jos, Nigeria. Front. Microbiol..

[27] Saliu E.-M., Vahjen W., Zentek J. (2017). Types and prevalence of extended-spectrum beta-lactamase producing Enterobacteriaceae in poultry. Anim. Health Res. Rev..

[28] Nhung N.T., Chansiripornchai N., Carrique-Mas J.J. (2017). Antimicrobial Resistance in Bacterial Poultry Pathogens: A Review. Front. Vet. Sci..

[29] Stromberg Z.R., Johnson J.R., Fairbrother J.M., Kilbourne J., Van Goor A., Curtiss R., Mellata M. (2017). Evaluation of *Escherichia coli* isolates from healthy chickens to determine their potential risk to poultry and human health. PLoS ONE.

[30] Hamelin K., Bruant G., El-Shaarawi A., Hill S., Edge T.A., Fairbrother J., Harel J., Maynard C., Masson L., Brousseau R. (2007). Occurrence of Virulence and Antimicrobial Resistance Genes in *Escherichia coli* Isolates from Different Aquatic Ecosystems within the St. Clair River and Detroit River Areas. Appl. Environ. Microbiol..

[31] Foster-Nyarko E., Cottingham H., Wick R.R., Judd L.M., Lam M.M.C., Wyres K.L., Stanton T.D., Tsang K.K., David S., Aanensen D.M. (2023). Nanopore-only assemblies for genomic surveillance of the global priority drug-resistant pathogen, *Klebsiella pneumoniae*. Microb. Genom..

[32] Neal-McKinney J.M., Liu K.C., Lock C.M., Wu W.H., Hu J. (2021). Comparison of MiSeq, MinION, and hybrid genome sequencing for analysis of *Campylobacter jejuni*. Sci. Rep..

[33] Oh S., Nam S.K., Chang H.E., Park K.U. (2022). Comparative Analysis of Short- and Long-Read Sequencing of Vancomycin-Resistant *Enterococci* for Application to Molecular Epidemiology. Front. Cell. Infect. Microbiol..

[34] Griswold T., Kapsak C., Chen J.C., Den Bakker H.C., Williams G., Kelley A., Vidyaprakash E., Katz L.S. (2021). SneakerNet: A modular quality assurance and quality check workflow for primary genomic and metagenomic read data. J. Open Source Softw..

[35] Centers for Disease Control and Prevention (2019). “Laboratory Standard Operating Procedure for Whole Genome Sequencing On Miseq.” Protocol PNL38. US Centers for Disease Control and Prevention. https://www.aphl.org/programs/global_health/Documents/PNL38_WGS%20on%20MiSeq%20SOP_v4.pdf.

[36] Kim D., Song L., Breitwieser F.P., Salzberg S.L. (2016). Centrifuge: Rapid and sensitive classification of metagenomic sequences. Genome Res..

[37] Afgan E., Baker D., Batut B., van den Beek M., Bouvier D., Čech M., Chilton J., Clements D., Coraor N., Grüning B.A. (2018). The Galaxy platform for accessible, reproducible and collaborative biomedical analyses: 2018 update. Nucleic Acids Res..

[38] Kapsak C., Katz L. NanoporeWorkflow v0.4.4. https://github.com/kapsakcj/nanoporeWorkflow.

[39] Wick R. Filtlong. https://github.com/rrwick/Filtlong.

[40] De Coster W., D’Hert S., Schultz D.T., Cruts M., Van Broeckhoven C. (2018). NanoPack: Visualizing and processing long-read sequencing data. Bioinformatics.

[41] Kolmogorov M., Yuan J., Lin Y., Pevzner P.A. (2019). Assembly of long, error-prone reads using repeat graphs. Nat. Biotechnol..

[42] Li H. (2018). Minimap2: Pairwise alignment for nucleotide sequences. Bioinformatics.

[43] Vaser R., Sovic I., Nagarajan N., Mile Š. (2017). Fast and accurate de novo genome assembly from long uncorrected reads. Genome Res..

[44] Oxford Nanopore Technologies LLC Medaka v1.2.3. https://github.com/nanoporetech/medaka.

[45] Krawczyk P.S., Lipinski L., Dziembowski A. (2018). PlasFlow: Predicting plasmid sequences in metagenomic data using genome signatures. Nucleic Acids Res..

[46] Katz L. SneakerNet. https://github.com/lskatz/SneakerNet.

[47] Bolger A.M., Lohse M., Usadel B. (2014). Trimmomatic: A flexible trimmer for Illumina sequence data. Bioinformatics.

[48] Ewels P., Magnusson M., Lundin S., Käller M. (2016). MultiQC: Summarize analysis results for multiple tools and samples in a single report. Bioinformatics.

[49] Wood D.E., Salzberg S.L. (2014). Kraken: Ultrafast metagenomic sequence classification using exact alignments. Genome Biol..

[50] Seemann T. Shovill. https://github.com/tseemann/shovill.

[51] Souvorov A., Agarwala R., Lipman D.J. (2018). SKESA: Strategic k-mer extension for scrupulous assemblies. Genome Biol..

[52] Hyatt D., Chen G., Locascio P.F., Land M.L., Larimer F.W., Hauser L.J. (2010). Prodigal: Prokaryotic gene recognition and translation initiation site identification. BMC Bioinform..

[53] Seemann T. (2014). Prokka: Rapid prokaryotic genome annotation. Bioinformatics.

[54] Wick R.R., Judd L.M., Gorrie C.L., Holt K.E. (2017). Unicycler: Resolving bacterial genome assemblies from short and long sequencing reads. PLoS Comput. Biol..

[55] Larsen M.V., Cosentino S., Lukjancenko O., Saputra D., Rasmussen S., Hasman H., Sicheritz-Pontén T., Aarestrup F.M., Ussery D.W., Lund O. (2014). Benchmarking of methods for genomic taxonomy. J. Clin. Microbiol..

[56] Bortolaia V., Kaas R.S., Ruppe E., Roberts M.C., Schwarz S., Philippon A., Allesoe R.L., Rebelo A.R., Florensa A.F., Cattoir V. (2020). ResFinder 4.0 for predictions of phenotypes from genotypes. J. Antimicrob. Chemother..

[57] Joensen K.G., Scheutz F., Lund O., Hasman H., Kaas R.S., Nielsen E.M., Aarestrup M. (2014). Real-Time Whole-Genome Sequencing for Routine Typing, Surveillance, and Outbreak Detection of Verotoxigenic *Escherichia coli*. J. Clin. Microbiol..

[58] Carattoli A., Zankari E., García-fernández A., Larsen V., Lund O., Villa L., Aarestrup M., Hasman H. (2014). In Silico Detection and Typing of Plasmids Using PlasmidFinder and Plasmid Multilocus Sequence Typing. Antimicrob. Agents Chemother..

[59] Franz E., Veenman C., van Hoek A.H.A.M., Husman A.D.R., Blaak H. (2015). Pathogenic *Escherichia coli* producing Extended-Spectrum β-Lactamases isolated from surface water and wastewater. Sci. Rep..

[60] Sarowska J., Koloch B.F., Kmiecik A.J., Madrzak M.F., Ksiazczyk M., Ploskonska G.B., Krol I.C. (2019). Virulence factors, prevalence and potential transmission of extraintestinal pathogenic *Escherichia coli* isolated from different sources: Recent reports. Gut Pathog..

[61] Page A.J., Cummins C.A., Hunt M., Wong V.K., Reuter S., Holden M.T.G., Fookes M., Falush D., Keane J.A., Parkhill J. (2015). Sequence analysis Roary: Rapid large-scale prokaryote pan genome analysis. Bioinformatics.

[62] Stamatakis A. (2014). RAxML version 8: A tool for phylogenetic analysis and post-analysis of large phylogenies. Bioinformatics.

[63] Letunic I., Bork P. (2019). Interactive Tree of Life (iTOL) v4: Recent updates and new developments. Nucleic Acids Res..

